# An Endometrial Thickness < 8 mm Was Associated With a Significantly Increased Risk of EP After Freeze-Thaw Transfer: An Analysis of 5,960 Pregnancy Cycles

**DOI:** 10.3389/fendo.2022.884553

**Published:** 2022-06-16

**Authors:** Ying Zhao, Dong’e Liu, Nenghui Liu, Yumei Li, Zhongyuan Yao, Fen Tian, Aizhuang Xu, Yanping Li

**Affiliations:** ^1^ Department of Reproductive Medicine, Xiangya Hospital, Central South University, Changsha, China; ^2^ Clinical Research Center for Women’s Reproductive Health in Hunan Province, Hunan, China

**Keywords:** ectopic pregnancy, EMT, endometrial type, freeze-thaw cycles, cleavage stage embryo, blastocyst

## Abstract

**Introduction:**

Endometrium characteristics that are most likely to induce ectopic pregnancy were investigated on the basis of the data of 5,960 pregnant freeze-thaw cycles.

**Methods:**

A total of 5,960 pregnancy cycles after freeze-thaw embryos transfer were included, with the number of intrauterine and ectopic pregnancies being 5,777 and 183, respectively. Ectopic pregnancy was the primary outcome. Endometrial thickness was the main measured variable. The risk factors of ectopic pregnancy were eventually determined based on univariate analysis and subsequent multiple-stepwise logistic regression analysis.

**Results:**

1. After adjusting for confounders, endometrial thickness could independently predict ectopic pregnancy. The adjusted odd ratios for women with endometrial thickness in the ranges of < 8 mm, 8–9.9 mm, and 10–11.9 mm were 3.270 [95% confidence interval (CI), 1.113–9.605, *P* = 0.031], 2.758 (95% CI, 0.987–7.707, *P* = 0.053), and 1.456 (95% CI, 0.502–4.225, *P* = 0.489), respectively, when compared with those having an endometrial thickness of 12–13.9 mm. 2. Endometrial type and preparation protocol were however not identified as risk factors for ectopic pregnancy.

**Discussion:**

1. After freeze-thaw embryo transfer, risks of ectopic pregnancy were significantly higher when the endometrial thickness was < 8 mm. 2. A thin endometrial thickness could be linked with abnormal endometrial peristaltic waves or abnormal endometrial receptivity. 3. Adequate attention should therefore be paid to patients with a thin endometrial thickness to prevent EP or to achieve early diagnosis during the peri-transplantation period.

## Background

Embryo implantation outside the uterine cavity represents an abnormal and dangerous form of pregnancy referred to as ectopic pregnancy (EP) (1), and according to reports, this condition is responsible for less than 1% of all maternal deaths in developing countries, with the figures rising to 5% for developed ones (2).

Assisted reproductive technology (ART) can theoretically decrease the incidence of EP, as both fertilization and embryo transfer (ET) do not involve the fallopian tubes. However, the rate of EP is around 1%–2% in spontaneous pregnancy as compared to 1.4%–5.4% in the case of ART (3, 4), and this can generally be attributed to risk factors such as low BMI (5), fresh ETs compared with freeze-thaw cycles (6–11), transfer of multiple embryos (12), and tubal factor infertility (TFI) (12–17). Similarly, the developmental stage of transferred embryos may be an important factor although its impact on EP incidence remains debatable (18–21).

In addition, few studies have also investigated the association between endometrial thickness (EMT) and EP occurrence after ART treatment, but there is no consensus on which EMT is applicable for EP prevention. Thus, identifying endometrial risk factors of EP and endometrial characteristics that could potentially predict EP after ART can be important.

## Methods

### Definition of Clinical Outcomes

Intrauterine pregnancy (IUP), as confirmed by ultrasonic assessment, was defined as the condition when at least one gestational sac was present in the uterine cavity. In contrast, when the gestational sac/mass was observed on the outside of the uterine cavity after ultrasonography, it was considered as a case of EP. Finally heterotopic pregnancy (HP) was defined as the simultaneous occurrence of an intrauterine sac and EP. Twelve days after ET, all patients underwent blood tests to assess levels of β-human chorionic gonadotropin (β-hCG), with those having β-hCG levels below 5 IU/L and above 15 IU/L being considered as negative and positive for pregnancy, respectively. In addition, patients were classified as indeterminate if their blood β-hCG levels were in the range of 5–15 IU/L, but if the levels increased after 48 h, they were subsequently classified as positive. All positive women received transvaginal ultrasound examinations 4–5weeks after ET. As the primary purpose of this analysis was to investigate the endometrial variables associated with EP risks, heterotopic pregnancies were excluded.

### Study Design and Patients

Between January 2014 and November 2021, the women underwent freeze-thaw ET at the Reproductive Medicine Centre of Xiangya hospital, Central South University (Changsha, China). Before ART procedure, ultrasonic examinations were performed at least twice at different periods of the menstrual cycles, with patients accepting further examination and treatment by hysteroscope if cavity abnormalities were found.

**Figure 1:**
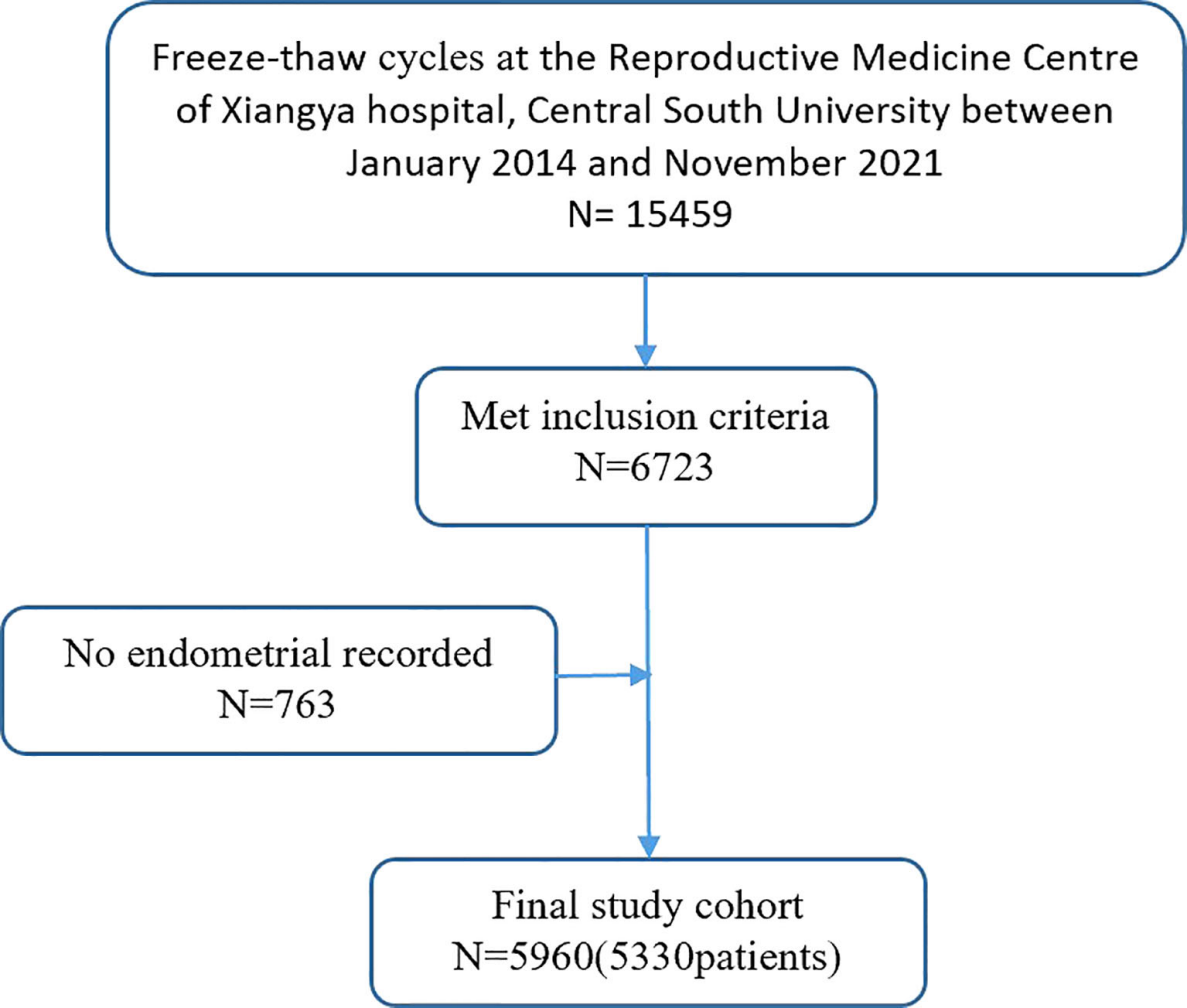
Flowchart of patients.

Patient data were collected from medical records. **Figure 1** presents a simplified selection process for IVF cycles. The following exclusion criteria was also applied: (1) non-pregnant cycles; (2) biochemical pregnancy; (3) HP; (4) cesarean scar pregnancy; (5) fresh embryos; (6) donor oocytes cycles; (7) presence of a known uterine anomaly; and (8) unrecorded endometrial data. As a result, from the initial 15,459 freeze-thaw ETs, 5,777 cycles were identified as IUP and 183 as EP.

EP was the primary outcome measured, whereas EMT was the main variables. Demographic data included patients age, previous history of EP, infertility type, infertility duration (years), etiology of infertility, parity, body mass index (BMI), number of embryos transferred, the developmental stage of the transferred embryos, and endometrial preparation protocol, as evaluated by the patient’s treating doctor. Patients were considered as presenting TFI if they reported any of the following: previous EP, previous salpingectomy, hydrosalpinx, or tubal scarring including occlusion. Similarly, women were diagnosed with polycystic ovary syndrome if they presented any two of the following characteristics: PCOM, ovulatory dysfunction, and clinical and/or biochemical hyperandrogenism. Finally, diminished ovarian reserve was diagnosed when women returned an abnormal ovarian reserve test (i.e., AMH < 0.5–1.1 ng/ml or antral follicular count (AFC) < 5–7 follicles) or presented any of the risk factors for POR. The follow-up rate was 100% in this study.

### Assessment of Primary Exposure

On the day of hCG administration for the natural and induced ovulation cycles, EMTs were monitored by transvaginal ultrasound scans. On the other hand, when providing hormone replacement cycle (HRT) and HRT combined with downregulation, EMT was measured when the last ultrasound exam was performed before progesterone (P) administration starting. EMT was measured on sagittal view, with the maximal anteroposterior thickness used by transvaginal sonography (22). According to the Gonen system (23), the endometrial morphology can be classified into three types: type A, trilaminar pattern (a triple-line pattern) that consist of hypoechoic inner layers, hyperechoic middle and outer layers, and evident echo at the intrauterine center line; type B, the echo of the endometrium is relatively homogeneous and hyperechoic, with a clear interface between muscular layers and the endometrium but unclear endometrial layers and obscure intrauterine center line echo; type C, the echo of the endometrium is homogeneous hyperechoic without intrauterine center line. EMTs were measured by six physicians who had received standardized training.

### Endometrial Preparation Protocol

Four protocols were available to prepare the endometrium for freeze-thaw transfer (FET): natural cycles, induced ovulation cycles, hormone replacement therapy cycles, and HRT after downregulation cycles. Natural cycles were used for patients who had regular ovulation cycles, whereas the other three were used for those without regular ovulation cycles.

In an induced ovulation cycle, follicular growth was induced as from the third day of menstruation by orally administering 2.5 mg of letrozole daily for 5 days. Then, as from the 10th day, transvaginal ultrasound exams were performed while monitoring the level of serum estrogen. In this case, if the diameter of the dominant follicle was found to be < 10 mm, then a daily injection of 37.5–75 IU of hMG was performed until the follicle’s diameter ≥ 17 mm. However, no injection was given if the follicle diameter was > 14 mm. In both natural and induced ovulation cycles, once the dominant follicle’s average diameter was > 17 mm and, at the same time, other conditions such as an EMT of >7 mm, P of < 1 ng/ml, and E_2_ of > 150 pg/ml were met, two types of treatment was administered based on LH levels in the serum. In cases where LH levels were < 20 mIU/ml, a night injection of 10,000 IU of hCG was provided, with P subsequently administered after 3 days. Embryos at the cleavage stage were then transferred 5 days after hCG had been administered, whereas for blastocysts, transfer was made after 7 days. When LH levels were > 20 mIU/ml, hCG was injected in the afternoon, with P administered after 2 days. Transfer of embryos at the cleavage stage was then performed 4 days after hCG injection, whereas for blastocysts, transfer was made after 6 days. For the HRT cycle, a dose of 4-6 mg of E_2_ was administered daily as from the third day of the period. Transvaginal ultrasound scans were then performed after 6 and 12 days. In this case, if an EMT >7 mm was observed along with the absence of ovulation signs or a main follicle, then the luteal phase was supported with a dose of 200 mg of oral progesterone capsules (Qining) once a day, accompanied with a 200-mg dose of vaginal micronized progesterone (Utrogestan) three times per day for 75 days. The course of E_2_ treatment did not last for less than 12 days or more than 21 days as it was previously shown that extended exposure to E_2_ could decrease the rates of live birth and clinical pregnancy (24). Finally, cleavage-stage ETs or blastocysts transfer were performed on the third or the fifth day, respectively. HRT after downregulation cycles differed from HRT in the use of one to two doses of 3.75 mg of GnRH-a during the early follicular phase before using E_2_.

### Statistical Analyses

Variables for EP were selected based on previous literature (25–27) and availability of data. SPSS version 23 (IBM) was used for data analysis and for quantitative data, the median (quartile interval), and mean ± SD were used to, respectively, describe normal and non-normal distributions. In the case of categorical data, the proportion of cases was presented as percentages.

For univariate comparisons, Pearson chi-square test and Mann–Whitney U test were used for categorical variables and non-normal distribution respectively. The risk factors linked with EP were then determined on the basis of stepwise multiple logistic regression analysis, with a receiver operating characteristic (ROC) curve generated for the predictors of EP. Finally, area under curve (AUC)–based validation of the model was performed. For analysis, differences with *P*-values < 0.05 were considered to be of statistical significance.

## Results

**Figure 1** shows the flowchart for cycle admission. This study included a total of 5,960 freeze-thaw cycles, including 5,777 IUP and 183 EP cycles.

The baseline characteristics were as shown in **Table 1**. The women’s age, BMI, infertility type and duration, endometrial type, male factor infertility, polycystic ovary syndrome, diminished ovarian reserve, intrauterine adhesions, scarred uterus, and endometrial preparation regime were not significantly different between two groups. Significant differences were, however, found for previous history of EP, the developmental stage of the transferred embryos, the number of embryos transferred, gravidity, TFI, and endometriosis (P < 0.05).

**Table 1:** Comparison of the baseline data of two groups patients.

Variables Intrauterine	Ectopic	Intrauterine	*P* value
	pregnancy,	pregnancy,	
	n = 5,777	n = 183	
Age (years)	31 (28,34)	30 (27,34)	0.059
Body mass index (kg/m^2^)	21.48 (19.70,23.63)	20.93 (19.55,23.41)	0.175
Infertility type			0.135
Primary sterility	2913 (50.4%)	82 (44.8%)	
Secondary sterility	2864 (49.6%)	101 (55.2%)	
Infertility duration (years)	4 (2,6)	4 (2,7)	0.479
Previous history of ectopic pregnancy			0.000*
=0	4905 (84.9%)	137 (74.9%)	
≥1	866 (15.1%)	46 (25.1%)	
Gravidity			0.014*
=0	3022 (52.3%)	79 (43.2%)	
≥1	2750 (47.7%)	104 (56.8%)	
Tubal factor infertility			0.001*
Yes	4860 (84.1%)	171 (93.4%)	
No	917 (15.9%)	12 (6.6%)	
Male factor infertility			0.327
Yes	1807 (31.3%)	51 (27.9%)	
No	3970 (68.7%)	132 (72.1%)	
Endometriosis			0.019*
Yes	279 (4.8%)	2 (1.1%)	
No	5498 (95.2%)	181 (98.9%)	
Polycystic ovary syndrome			0.962
Yes	828 (14.3%)	26 (14.2%)	
No	4949 (85.7%)	157 (85.8%)	
Diminished ovarian reserve			0.455
Yes	467 (8.1%)	12 (6.6%)	
No	5310 (91.9%)	171 (93.4%)	
IUA			0.222
Yes	229 (4.0%)	4 (2.2%)	
No	5548 (96.0%)	179 (97.8%)	
Scarred uterus			0.147
Yes	452 (7.8%)	9 (4.9%)	
No	5325 (92.2%)	174 (95.1%)	
No. of embryos transferred			0.000*
1	1717 (29.7%)	31 (16.9%)	
2	4047 (70.1%)	150 (82.0%)	
3	13 (0.2%)	2 (1.1%)	
Endometrial thickness (mm)	9.50 (8.50,10.70)	8.60 (8.10,9.60)	0.000*
Endometrial thickness (mm)			0.000*
<8	675 (11.7%)	37 (20.2%)	
8–9.9	2818 (48.8%)	115 (62.8%)	
10–11.9	1724 (29.8%)	27 (14.8%)	
12–13.9	444 (7.7%)	4 (2.2%)	
≥14	116 (2.0%)	0 (0%)	
Endometrial type			0.396
A	1974 (34.2%)	70 (38.3%)	
B	3270 (56.6%)	100 (54.6%)	
C	533 (9.2%)	13 (7.1%)	
Endometrial Preparation Regime			0.309
Hormone replacement therapy	3130 (54.2%)	111 (60.7%)	
Hormone replacement therapy after downregulation	407 (7.0%)	9 (4.9%)	
Induced ovulation cycle	333 (5.8%)	8 (4.4%)	
Natural cycle	1907 (33.0%)	55 (30.1%)	
Embryo stage			0.000*
Cleavage stage embryo	3301 (57.1%)	147 (80.3%)	
Blastocyst	2476 (42.9%)	36 (19.7%)	

EP, ectopic pregnancy; IUP, intrauterine pregnancy; BMI, body mass index. *The difference is significant between groups.

EMTs of women with EP were significantly thinner in comparison with IUP patients (*P <*0.001). In addition, the EP rate in women with EMT < 8 mm (5.2%) was significantly higher than for those with an EMT of 8–9.9 mm (3.9%), 10–11.9 mm (1.5%), 12–13.9 mm (0.9%), and ≥ 14 mm (0%) (*P* < 0.001). In fact, none of the patients with an EMT of ≥ 14 mm developed EP in subsequent analysis and as such, those with EMT of 12–13.9 mm were selected as the reference group.

**Table 2** shows the results of the univariate analysis. Compared with an EMT of 12–13.9 mm as the reference, the risk of EP in patients with an EMT of < 8 mm was fivefold (OR, 6.084; 95% CI 2.154–17.189; *P* = 0.001), with the risk decreasing to threefold in the case of those with an EMT of 8–9.9 mm (OR, 4.530; 95% CI, 1.663–12.337; *P* = 0.003). However, the EP risk for women with an EMT of 10–11.9 mm was not statistically different from that of the reference group (i.e., 12–13.9 mm) (OR, 1.738; 95% CI, 0.605–4.994; P = 0.304).

**Table 2:** Factors related to ectopic pregnancy based on univariate analysis.

Predictor variables	Odds ratio	95% confidence interval	*P* value
Age (years)	0.974	0.943, 1.007	0.121
BMI (kg/m^2^)	1.001	1.000, 1.002	0.043*
Infertility type			
Primary infertile	1	——	——
Secondary sterility	1.253	0.932, 1.684	0.135
Infertility duration (years)	1.009	0.967, 1.052	0.680
Previous history of ectopic pregnancy			
=0	1	——	——
≥1	1.902	1.351, 2.676	0.000*
Gravidity			
=0	1	——	——
≥1	0.691	0.514, 0.930	0.015*
Tubal factor infertility			
Yes	2.689	1.491, 4.848	0.001*
No	1	——	——
Male factor infertility			0.327
Yes	0.849	0.612, 1.178	
No	1	——	——
Endometriosis			0.033*
Yes	0.218	0.054, 0.882	
No	1	——	——
Polycystic ovary syndrome			0.962
Yes	0.990	0.649, 1.509	
No	1	——	——
Diminished ovarian reserve			0.456
Yes	0.798	0.441, 1.444	
No	1	——	——
IUA			0.229
Yes	0.541	0.199, 1.471	
No	1	——	——
Scarred uterus			0.151
Yes	0.609	0.310, 1.199	
No	1	——	——
No. of embryos transferred			0.000*
=1	1	——	——
=2	2.053	1.389, 3.034	0.000*
=3	8.521	1.844, 39.371	0.006*
Embryo stage			
Cleavage stage embryo	1	——	——
Blastocyst	0.326	0.226, 0.472	0.000*
Endometrial thickness (mm)	0.700	0.629, 0.779	0.000*
Endometrial thickness (mm)			0.000*
12-13.9	1	——	——
<8	6.084	2.154, 17.189	0.001*
8–9.9	4.530	1.663, 12.337	0.003*
10–11.9	1.738	0.605, 4.994	0.304
≥14	0.000	0.000, 0.000	0.996
Endometrial type			0.398
A	1	——	——
B	0.862	0.632, 1.176	0.350
C	0.688	0.378, 1.253	0.221
Ovarian stimulation protocol			0.314
Hormone replacement therapy	1	——	——
Hormone replacement therapy after downregulation	0.624	0.314, 1.240	0.178
Induced ovulation cycle	0.677	0.328, 1.401	0.293
Natural cycle	0.813	0.586, 1.129	0.217

*The difference is significant between the two groups.

The risk factors for EP were a small BMI, an EP history, TFI, multiple embryos transfer, and transfer of embryos at the cleavage stage. In contrast, history of IUP and endometriosis infertility was protective against EP, whereas endometrial preparation regime was not identified as a risk factor for EP.

For multivariate stepwise regression analysis, all variables with a P-value of < 0.1 during univariate analysis were included, with the results shown in **Table 3**.

**Table 3:** Factors associated with ectopic pregnancy based on stepwise multiple regression analysis.

Predictor variables	Odds ratio	95%	*P* value
		confidence	
		interval	
Previous history of ectopic pregnancy			
=0	1	——	——
≥1	1.573	1.102, 2,243	0.012*
BMI (kg/m^2^)	1.001	1.000, 1.002	0.034*
Tubal factor infertility			
Yes	2.221	1.191, 4.144	0.012*
No	1	——	——
Embryo stage			
Cleavage stage embryo	1	——	——
Blastocyst	0.451	0.297, 0.683	0.000*
Endometrial thickness (mm)			0.021*
12–13.9	1	——	——
<8	3.270	1.113, 9.605	0.031*
8–9.9	2.758	0.987, 7.707	0.053
10–11.9	1.456	0.502, 4.225	0.489
≥14	0.000	0.000, 0.000	0.996

* The difference is significant between groups.

A small BMI (aOR, 1.001; 95% CI, 1.000–1.002; P = 0.034) and blastocyst transfer (aOR, 0.451; 95% CI, 0.297–0.683; *P* < 0.001) were protective factors against EP after FET cycles. On the other hand, TFI (aOR, 2.221; 95% CI, 1.191–4.144; P = 0.012), a previous history of EP (aOR, 1.573; 95% CI, 1.102–2.243; P = 0.012) and an EMT of < 8 mm (aOR, 3.270; 95% CI, 1.113–9.605; P = 0.031) before P administration were found to independently predict EP. An EMT of 8–9.9 mm (aOR, 2.758; 95% CI, 0.987–7.707; P = 0.053) and 10–11.9 mm (aOR, 1.456; 95% CI, 0.502–4.225; P = 0.489) was not significantly linked with EP occurrence.

**Figure 2:**
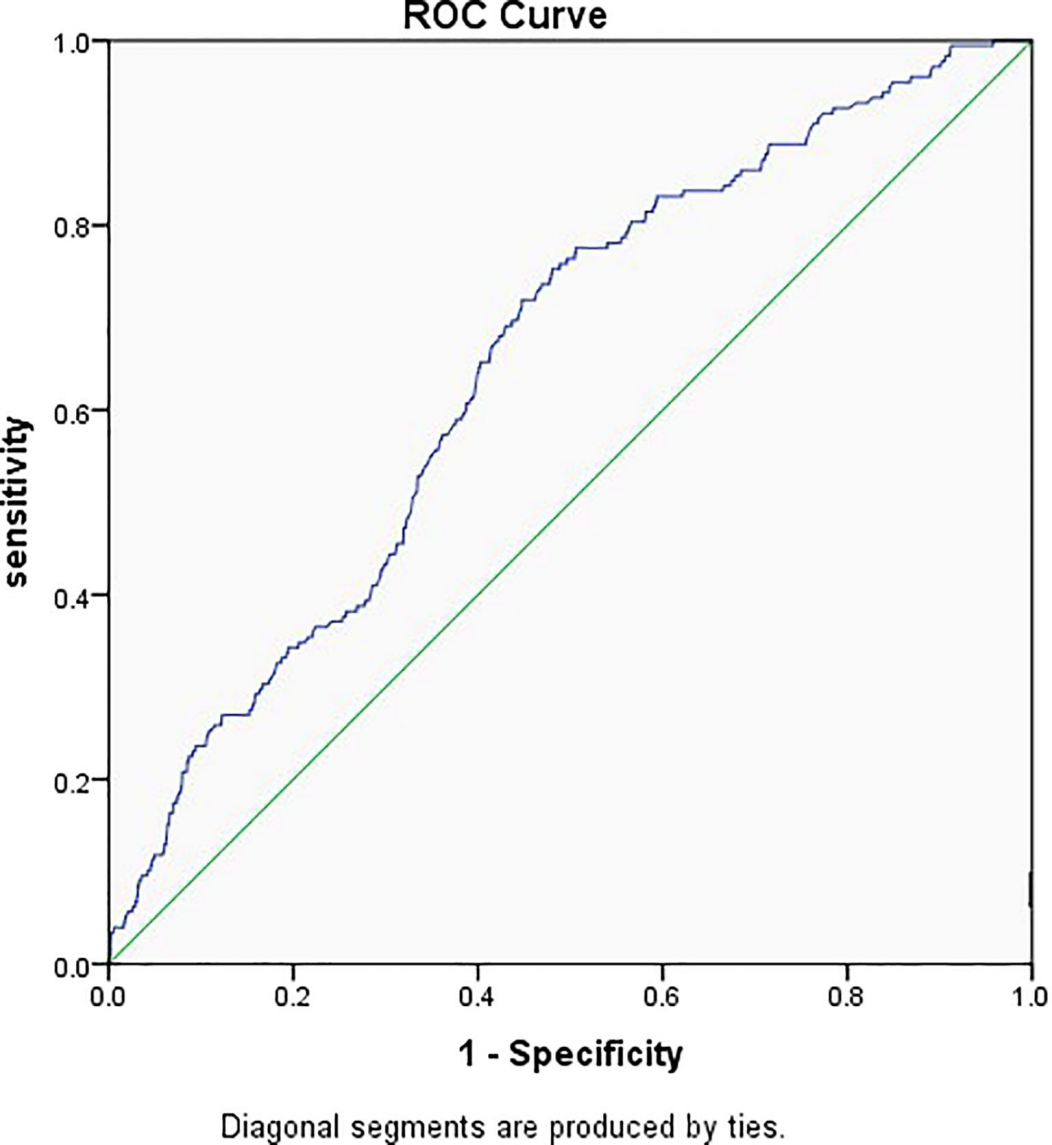
Receiver operator characteristic curve of the embryo stage, BMI, previous history of ectopic pregnancy, and tubal factor infertility. Diagonal segments were produced by ties and the area under the curve was 0.651.

**Figure 3:**
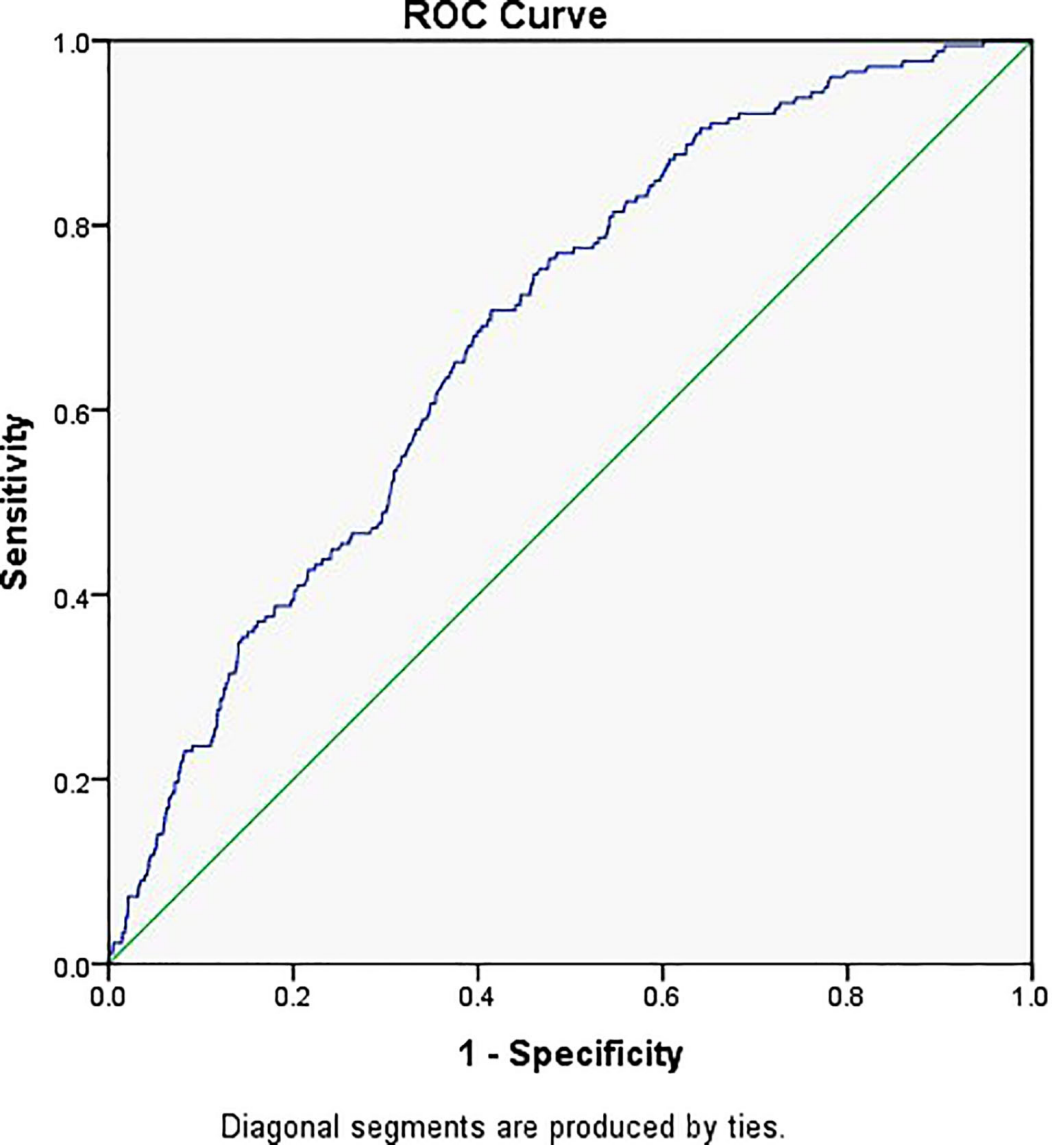
Receiver operator characteristic curve of the embryo stage, BMI, tubal factor infertility, previous history of ectopic pregnancy, and EMT. Diagonal segments were produced by ties and the area under the curve was 0.686.

**Figure 2** shows the prediction model for EP, with the embryo stage, BMI, previous history of EP, and TFI as the variables. The suitability of the model was assessed with an ROC curve for which the area under the curve was 0.651 (95% CI 0.612–0.689, *P* < 0.001). After including EMT as one of the variables of the model, the AUC increased to 0.686 (95% CI 0.650–0.722, *P* < 0.001) (**Figure 3**).

## Discussion

This retrospective analysis of frozen ET cycles sought to investigate the possible link between EMT and EP after freeze-thaw transfer.

In this study, TFI increased the risk of EP by more than twofold, with the results supported by previous studies (12, 13, 15, 26, 28–31). Similarly, women with low BMI were more likely to develop EP after ART, with this outcome being consistent with a previous research (5). As for multiple embryos transfer, although some studies (26, 28, 32) reported that it was risk factor for EP, another one considered that it actually had no impact (29). In fact, previous history of EP (29, 33) is similarly debatable. In the present work, multiple embryos transfer was not identified as a risk factor.

Consistent with some previous studies (34–37), transferring embryos at cleavage stage was a risk factor of EP in the current study. Embryos at this stage could be more prone to “traveling around”, unlike blastocysts that tend to immediately seek contact and attachment. Furthermore, a study has shown that decreased uterine contractions on the fifth day could also lower EP rate after blastocysts transfer (19). However, some studies (19, 38–41) do suggest that EP occurrence was not influenced by the stage of embryos. In fact, some reports even suggest that blastocysts transfer could actually heighten EP risks due to potentially higher implantation rates of each blastocyst (20, 25).

These inconsistencies could be the result of variations between studies, especially in terms of patients’ age, sample sizes, evaluation system for embryos, and differences in blastocysts culture techniques between reproductive centers.

Few studies have examined the suitability of EMT during ART therapy for predicting EP, but the currently applicable cutoff value that link EMT and EP is controversial. One study in which fresh or frozen embryo cycles were not separately analyzed suggested that, prior to ET, an EMT > 12 mm protected against EP (29). In the same vein, another study reported increased risks of EP when the EMT was < 12 mm in the frozen embryo cycle (28). The current study indicated that an EMT of < 8 mm significantly increased risks of EP. Because it is clinically impractical to perform ET only if the EMT is greater than 12mm, it would therefore be more clinically meaningful to select an EMT of < 8 mm rather than 12 mm as the threshold for being considered a risk factor for EP.

It remains unclear why a thin endometrium increases risks of EP rates, with the reason likely to be complex. Many researchers have suggested that there is a relationship between EMT and uterine receptivity (42–47), and, as such, it is generally believed that thinner EMTs may lead to a poorer endometrial receptivity.

Differences in oxygen concentrations could also explain the link between a thin endometrium and EP. Indeed, a thin EMT would bring embryos quite close to the spiral arteries in the basal endometrium layer, thereby exposing them to high oxygen concentrations which are known to inhibit embryonic development (48).

Uterine peristalsis could be another factor that links EP incidence with a thin endometrium. Previous studies have reported that, compared with IUP, women diagnosed with EP experienced higher uterine peristaltic wave frequencies, but these differences were not statistically significant due to uneven distribution of the sample size (49). A previous research further showed the EMT thickness was positively associated with risks of placenta previa (50). In this case, the authors hypothesized that high EMTs were an indication of the amplitude and/or frequency of uterine peristalsis waves, which can push the embryos downward, dislodging them from their transferred location. Another study (51) found that, compared with natural cycles, controlled ovarian hyperstimulation cycles showed increased uterine waves from the cervix to the fundus but reduced ones from the fundus to the cervix. Therefore, although the results of that study suggest that the direction of uterine peristalsis could influence EP occurrence, yet this link would need to be confirmed through additional studies. Altogether, this study’s findings point out that uterine peristalsis from the fundus to the cervix is more likely to occur when the endometrium is thicker as the embryos are more easily implanted in the lower segment of the uterine cavity, resulting in a higher incidence of placenta previa (50) and, correspondingly, a lower incidence of EP. Future studies will focus on how different EMT are associated with the frequency, amplitude, and direction of endometrial peristalsis waves, including the correlation between EP occurrence and the presence of different types of endometrial peristalsis at the time of ET.

As EP risks were higher for patients with an EMT of < 8 mm, especially when the transferred embryos were in the cleavage stage or when multiple embryos were transferred, such patients should be given clear advice. Furthermore, it should be carefully decided whether frozen ET should be performed in the current cycle while routinely examining the endometrial peristaltic wave (49, 52) and providing appropriate treatments such as phloroglucinol (53) and atosiban (54) when necessary as these would help in preventing EP or diagnosing the condition at an early stage.

This study was not without limitations, with the first one being that it was a retrospective study. Chromosomal abnormalities of embryos as a potential risk factor for EP were also not included as not all embryos were tested for chromosomes before transfer, but it was previously reported (55, 56) that chromosomal abnormalities in embryos were not actively involved in the etiology of EP. In addition, the study contained a high rate of multiple embryos transfer. Some researchers (26, 28) believe that multiple embryos transfer is an independent risk factor for EP as well as HP, but this study did not find this variable to be an independent risk factor after multivariate regression analysis, consistent with a previous study (32). This could have been due to the exclusion of HP in the present research.

In short, the results can be very meaningful in the absence of a consensus about the optimal EMT, which can predict or prevent EP after ART.

## Conclusion

An EMT < 8 mm on the day of endometrial transformation was found to independently predict EP after freeze-thaw ET. Efforts to increase the EMT may reduce EP risks. It may also be necessary to perform endometrial peristaltic wave examination, endometrial receptivity testing as well as provide corresponding treatment in the peri-transfer period for patients with risk factors.

## Data Availability Statement

The raw data supporting the conclusions of this article will be made available by the authors, without undue reservation.

## Ethics Statement

Written informed consent was obtained from the individual(s) for the publication of any potentially identifiable images or data included in this article.

## Author Contributions

All authors contributed to the study conception and design. Material preparation, data collection, and analysis were performed by YZ. The first draft of the manuscript was written by YZ, and all authors commented on previous versions of the manuscript. All authors contributed to the article and approved the submitted version.

## Funding

This study was supported by the National Key Research and Developmental Program of China(2018 YFC1004800) and the National Natural Science Foundation of China (grant no. 8187061497).

## Conflict of Interest

The authors declare that the research was conducted in the absence of any commercial or financial relationships that could be construed as a potential conflict of interest.

## Publisher’s Note

All claims expressed in this article are solely those of the authors and do not necessarily represent those of their affiliated organizations, or those of the publisher, the editors and the reviewers. Any product that may be evaluated in this article, or claim that may be made by its manufacturer, is not guaranteed or endorsed by the publisher.

## References

[1] ACOG. Practice Bulletin No. 191 Summary: Tubal Ectopic Pregnancy. Obstet Gynecol (2018) 131(2):409–11. doi: 10.1097/aog.0000000000002499 29370045

[2] Khan K Wojdyla D Say L Gülmezoglu A Van Look P. WHO Analysis of Causes of Maternal Death: A Systematic Review. Lancet (London England) (2006) 367(9516):1066–74. doi: 10.1016/s0140-6736(06)68397-9 16581405

[3] Jurkovic D Wilkinson H. Diagnosis and Management of Ectopic Pregnancy. BMJ (Clinical Res) (2011) 342:d3397. doi: 10.1136/bmj.d3397 21665933

[4] Muller V Makhmadalieva M Kogan I Fedorova I Lesik E Komarova E. Ectopic Pregnancy Following *In Vitro* Fertilization: Meta-Analysis and Single-Center Experience During 6 Years. Gynecol Endocrinol Off J Int Soc Gynecol Endocrinol (2016) 32:69–74. doi: 10.1080/09513590.2016.1232550 27759446

[5] Cai J Liu L Jiang X Li P Sha A Ren J. Low Body Mass Index is Associated With Ectopic Pregnancy Following Assisted Reproductive Techniques: A Retrospective Study. BJOG Int J Obstet Gynecol (2021) 128(3):540–50. doi: 10.1111/1471-0528.16378 32575153

[6] Huang B Hu D Qian K Ai J Li Y Jin L. Is Frozen Embryo Transfer Cycle Associated With a Significantly Lower Incidence of Ectopic Pregnancy? An Analysis of More Than 30,000 Cycles. Fertil Steril (2014) 102(5):1345–9. doi: 10.1016/j.fertnstert.2014.07.1245 25241365

[7] Shapiro B Daneshmand S De Leon L Garner F Aguirre M Hudson C. Frozen-Thawed Embryo Transfer is Associated With a Significantly Reduced Incidence of Ectopic Pregnancy. Fertil Steril (2012) 98(6):1490–4. doi: 10.1016/j.fertnstert.2012.07.1136 22925683

[8] Shapiro B Daneshmand S Garner F Aguirre M Hudson C Thomas S. Evidence of Impaired Endometrial Receptivity After Ovarian Stimulation for *In Vitro* Fertilization: A Prospective Randomized Trial Comparing Fresh and Frozen-Thawed Embryo Transfer in Normal Responders. Fertil Steril (2011) 96(2):344–8. doi: 10.1016/j.fertnstert.2011.05.050 21737072

[9] Ishihara O Kuwahara A Saitoh H. Frozen-Thawed Blastocyst Transfer Reduces Ectopic Pregnancy Risk: An Analysis of Single Embryo Transfer Cycles in Japan. Fertil Steril (2011) 95(6):1966–9. doi: 10.1016/j.fertnstert.2011.02.015 21377154

[10] Polyzos N Devroey P. Significantly Lower Ectopic Pregnancy Rates After Frozen Embryo Transfer: Implications Toward Segmentation of *In Vitro* Fertilization Treatment. Fertil Steril (2012) 98(6):1419–20. doi: 10.1016/j.fertnstert.2012.08.044 22999794

[11] Londra L Moreau C Strobino D Garcia J Zacur H Zhao Y. Ectopic Pregnancy After *In Vitro* Fertilization: Differences Between Fresh and Frozen-Thawed Cycles. Fertil Steril (2015) 104(1):110–8. doi: 10.1016/j.fertnstert.2015.04.009 25956363

[12] Clayton H Schieve L Peterson H Jamieson D Reynolds M Wright V. Ectopic Pregnancy Risk With Assisted Reproductive Technology Procedures. Obstet Gynecol (2006) 107(3):595–604. doi: 10.1097/01.Aog.0000196503.78126.62 16507930

[13] Strandell A Thorburn J Hamberger L. Risk Factors for Ectopic Pregnancy in Assisted Reproduction. Fertil Steril (1999) 71(2):282–6. doi: 10.1016/s0015-0282(98)00441-5 9988399

[14] Pyrgiotis E Sultan K Neal G Liu H Grifo J Rosenwaks Z. Ectopic Pregnancies After *In Vitro* Fertilization and Embryo Transfer. J Assist Reprod Genet (1994) 11(2):79–84. doi: 10.1007/bf02215992 7529603

[15] Malak M Tawfeeq T Holzer H Tulandi T. Risk Factors for Ectopic Pregnancy After *In Vitro* Fertilization Treatment. J Obstet Gynecol Canada JOGC (2011) 33(6):617–9. doi: 10.1016/s1701-2163(16)34910-6 21846451

[16] Kazandi M Turan V. Ectopic Pregnancy; Risk Factors and Comparison of Intervention Success Rates in Tubal Ectopic Pregnancy. Clin Exp Obstet Gynecol (2011) 38(1):67–70. 21485731

[17] Refaat B Dalton E Ledger W. Ectopic Pregnancy Secondary to *In Vitro* Fertilisation-Embryo Transfer: Pathogenic Mechanisms and Management Strategies. Reprod Biol Endocrinol RB&E (2015) 13:30. doi: 10.1186/s12958-015-0025-0 25884617 PMC4403912

[18] Bu Z Xiong Y Wang K Sun Y. Risk Factors for Ectopic Pregnancy in Assisted Reproductive Technology: A 6-Year, Single-Center Study. Fertil Steril (2016) 106(1):90–4. doi: 10.1016/j.fertnstert.2016.02.035 27001382

[19] Milki A Jun S. Ectopic Pregnancy Rates With Day 3 Versus Day 5 Embryo Transfer: A Retrospective Analysis. BMC Pregnancy Childbirth (2003) 3(1):7. doi: 10.1186/1471-2393-3-7 14604439 PMC270025

[20] Keegan D Morelli S Noyes N Flisser E Berkeley A Grifo J. Low Ectopic Pregnancy Rates After *In Vitro* Fertilization: Do Practice Habits Matter? Fertil Steril (2007) 88(3):734–6. doi: 10.1016/j.fertnstert.2006.11.169 17316634

[21] Rosman E Keegan D Krey L Liu M Licciardi F Grifo J. Ectopic Pregnancy Rates After *In Vitro* Fertilization: A Look at the Donor Egg Population. Fertil Steril (2009) 92(5):1791–3. doi: 10.1016/j.fertnstert.2009.05.041 19524897

[22] Bredella M Feldstein V Filly R Goldstein R Callen P Genant H. Measurement of Endometrial Thickness at US in Multicenter Drug Trials: Value of Central Quality Assurance Reading. Radiology (2000) 217(2):516–20. doi: 10.1148/radiology.217.2.r00nv34516 11058654

[23] Zhao J Zhang Q Wang Y Li Y. Endometrial Pattern, Thickness and Growth in Predicting Pregnancy Outcome Following 3319 IVF Cycle. Reprod Biomed Online (2014) 29(3):291–8. doi: 10.1016/j.rbmo.2014.05.011 25070912

[24] Bourdon M Santulli P Kefelian F Vienet-Legue L Maignien C Pocate-Cheriet K. Prolonged Estrogen (E2) Treatment Prior to Frozen-Blastocyst Transfer Decreases the Live Birth Rate. Hum Reprod (Oxford England) (2018) 33(5):905–13. doi: 10.1093/humrep/dey041 29529202

[25] Chang H Suh C. Ectopic Pregnancy After Assisted Reproductive Technology: What are the Risk Factors? Curr Opin Obstet Gynecol (2010) 22(3):202–7. doi: 10.1097/GCO.0b013e32833848fd 20216415

[26] Perkins K Boulet S Kissin D Jamieson D. Risk of Ectopic Pregnancy Associated With Assisted Reproductive Technology in the United States, 2001-2011. Obstet Gynecol (2015) 125(1):70–8. doi: 10.1097/aog.0000000000000584 PMC4315158 25560107

[27] Li Z Sullivan E Chapman M Farquhar C Wang Y. Risk of Ectopic Pregnancy Lowest With Transfer of Single Frozen Blastocyst. Hum Reprod (Oxford England) (2015) 30(9):2048–54. doi: 10.1093/humrep/dev168 26202917

[28] Liu H Zhang J Wang B Kuang Y. Effect of Endometrial Thickness on Ectopic Pregnancy in Frozen Embryo Transfer Cycles: An Analysis Including 17,244 Pregnancy Cycles. Fertil Steril (2020) 113(1):131–9. doi: 10.1016/j.fertnstert.2019.09.003 31727414

[29] Liu X Qu P Bai H Shi W Shi J. Endometrial Thickness as a Predictor of Ectopic Pregnancy in 1125 *In Vitro* Fertilization-Embryo Transfer Cycles: A Matched Case-Control Study. Arch Gynecol Obstet (2019) 300(6):1797–803. doi: 10.1007/s00404-019-05353-z 31720777

[30] Lesny P Killick S Robinson J Maguiness S. Transcervical Embryo Transfer as a Risk Factor for Ectopic Pregnancy. Fertil Steril (1999) 72(2):305–9. doi: 10.1016/s0015-0282(99)00226-5 10439001

[31] Ribic-Pucelj M Tomazevic T Vogler A Meden-Vrtovec H. Risk Factors for Ectopic Pregnancy After *In Vitro* Fertilization and Embryo Transfer. J Assist Reprod Genet (1995) 12(9):594–8. doi: 10.1007/bf02212581 8580656

[32] Rombauts L McMaster R Motteram C Fernando S. Risk of Ectopic Pregnancy is Linked to Endometrial Thickness in a Retrospective Cohort Study of 8120 Assisted Reproduction Technology Cycles. Hum Reprod (Oxford England) (2015) 30(12):2846–52. doi: 10.1093/humrep/dev249 26428211

[33] Xu Z Yan L Liu W Xu X Li M Ding L. Effect of Treatment of a Previous Ectopic Pregnancy on *In Vitro* Fertilization-Intracytoplasmic Sperm Injection Outcomes: A Retrospective Cohort Study. Fertil Steril (2015) 104(6):1446–51.e1441-1443. doi: 10.1016/j.fertnstert.2015.08.034 26409152

[34] Fang C Huang R Wei L Jia L. Frozen-Thawed Day 5 Blastocyst Transfer is Associated With a Lower Risk of Ectopic Pregnancy Than Day 3 Transfer and Fresh Transfer. Fertil Steril (2015) 103(3):655–61.e653. doi: 10.1016/j.fertnstert.2014.11.023 25542820

[35] Li R Dong Y Guo Y Sun Y Su Y Chen F. Comparative Study of Pregnancy Outcomes Between Day 3 Embryo Transfer and Day 5 Blastocyst Transfer in Patients With Progesterone Elevation. J Int Med Res (2013) 41(4):1318–25. doi: 10.1177/0300060513489480 23812114

[36] Du T Chen H Fu R Chen Q Wang Y Mol B. Comparison of Ectopic Pregnancy Risk Among Transfers of Embryos Vitrified on Day 3, Day 5, and Day 6. Fertil Steril (2017) 108(1):108–16.e101. doi: 10.1016/j.fertnstert.2017.05.027 28602476

[37] Zhang B Cui L Tang R Ding L Yan L Chen Z. Reduced Ectopic Pregnancy Rate on Day 5 Embryo Transfer Compared With Day 3: A Meta-Analysis. PLos One (2017) 12(1):e0169837. doi: 10.1371/journal.pone.0169837 28121989 PMC5266274

[38] Smith L Oskowitz S Dodge L Hacker M. Risk of Ectopic Pregnancy Following Day-5 Embryo Transfer Compared With Day-3 Transfer. Reprod Biomed Online (2013) 27(4):407–13. doi: 10.1016/j.rbmo.2013.06.015 23953586

[39] Cheng L Lin P Huang F Kung F Chiang H Lin Y. Ectopic Pregnancy Following *In Vitro* Fertilization With Embryo Transfer: A Single-Center Experience During 15 Years. Taiwanese J Obstet Gynecol (2015) 54(5):541–5. doi: 10.1016/j.tjog.2015.08.004 26522107

[40] Wang E Kathiresan A Bresee C Greene N Alexander C Pisarska M. Abnormal Implantation After Fresh and Frozen *In Vitro* Fertilization Cycles. Fertil Steril (2017) 107(5):1153–8. doi: 10.1016/j.fertnstert.2017.03.012 PMC5628741 28433367

[41] Wang S Sun H. Blastocyst Transfer Ameliorates Live Birth Rate Compared With Cleavage-Stage Embryos Transfer in Fresh *In Vitro* Fertilization or Intracytoplasmic Sperm Injection Cycles: Reviews and Meta-Analysis. Yonsei Med J (2014) 55(3):815–25. doi: 10.3349/ymj.2014.55.3.815 PMC3990083 24719153

[42] McWilliams G Frattarelli J. Changes in Measured Endometrial Thickness Predict *In Vitro* Fertilization Success. Fertil Steril (2007) 88(1):74–81. doi: 10.1016/j.fertnstert.2006.11.089 17239871

[43] Amir W Micha B Ariel H Liat L Jehoshua D Adrian S. Predicting Factors for Endometrial Thickness During Treatment With Assisted Reproductive Technology. Fertil Steril (2007) 87(4):799–804. doi: 10.1016/j.fertnstert.2006.11.002 17207799

[44] Richter K Bugge K Bromer J Levy M. Relationship Between Endometrial Thickness and Embryo Implantation, Based on 1,294 Cycles of *In Vitro* Fertilization With Transfer of Two Blastocyst-Stage Embryos. Fertil Steril (2007) 87(1):53–9. doi: 10.1016/j.fertnstert.2006.05.064 17081537

[45] Zhang X Chen C Confino E Barnes R Milad M Kazer R. Increased Endometrial Thickness is Associated With Improved Treatment Outcome for Selected Patients Undergoing *In Vitro* Fertilization-Embryo Transfer. Fertil Steril (2005) 83(2):336–40. doi: 10.1016/j.fertnstert.2004.09.020 15705371

[46] Zenke U Chetkowski R. Transfer and Uterine Factors are the Major Recipient-Related Determinants of Success With Donor Eggs. Fertil Steril (2004) 82(4):850–6. doi: 10.1016/j.fertnstert.2004.03.057 15482759

[47] Kovacs P Matyas S Boda K Kaali S. The Effect of Endometrial Thickness on IVF/ICSI Outcome. Hum Reprod (Oxford England) (2003) 18(11):2337–41. doi: 10.1093/humrep/deg461 14585884

[48] Casper R. It's Time to Pay Attention to the Endometrium. Fertil Steril (2011) 96(3):519–21. doi: 10.1016/j.fertnstert.2011.07.1096 21880272

[49] Zhu L Che H Xiao L Li Y. Uterine Peristalsis Before Embryo Transfer Affects the Chance of Clinical Pregnancy in Fresh and Frozen-Thawed Embryo Transfer Cycles. Hum Reprod (Oxford England) (2014) 29(6):1238–43. doi: 10.1093/humrep/deu058 24664129

[50] Rombauts L Motteram C Berkowitz E Fernando S. Risk of Placenta Praevia is Linked to Endometrial Thickness in a Retrospective Cohort Study of 4537 Singleton Assisted Reproduction Technology Births. Hum Reprod (Oxford England) (2014) 29(12):2787–93. doi: 10.1093/humrep/deu240 25240011

[51] Zhu L Li Y Xu A. Influence of Controlled Ovarian Hyperstimulation on Uterine Peristalsis in Infertile Women. Hum Reprod (Oxford England) (2012) 27(9):2684–9. doi: 10.1093/humrep/des257 22798632

[52] Zhu L Xiao L Che H Li Y Liao J. Uterine Peristalsis Exerts Control Over Fluid Migration After Mock Embryo Transfer. Hum Reprod (Oxford England) (2014) 29(2):279–85. doi: 10.1093/humrep/det429 24277748

[53] Xu A Li Y Zhu L Tian T Hao J Zhao J. Inhibition of Endometrial Fundocervical Wave by Phloroglucinol and the Outcome of *In Vitro* Fertilization. Reprod Biol (2013) 13(1):88–91. doi: 10.1016/j.repbio.2013.01.165 23522076

[54] Buddhabunyakan N Sothornwit J Seejorn K Buppasiri P Salang L. Effects of Atosiban on Uterine Peristalsis Following Frozen Embryo Transfer: A Randomized Controlled Trial. Eur J Obstet Gynecol Reprod Biol (2021) 265:96–101. doi: 10.1016/j.ejogrb.2021.08.017 34478926

[55] Goddijn M van der Veen F Schuring-Blom G Ankum W Leschot N. Cytogenetic Characteristics of Ectopic Pregnancy. Hum Reprod (Oxford England) (1996) 11(12):2769–71. doi: 10.1093/oxfordjournals.humrep.a019207 9021388

[56] Coste J Fernandez H Joyé N Benifla J Girard S Marpeau L. Role of Chromosome Abnormalities in Ectopic Pregnancy. Fertil Steril (2000) 74(6):1259–60. doi: 10.1016/s0015-0282(00)01593-4 11119765

